# KML001 Displays Vascular Disrupting Properties and Irinotecan Combined Antitumor Activities in a Murine Tumor Model

**DOI:** 10.1371/journal.pone.0053900

**Published:** 2013-01-11

**Authors:** Chang Hoon Moon, Seung Ju Lee, Ho Yong Lee, Jong Cheol Lee, HeeJeong Cha, Wha Ja Cho, Jeong Woo Park, Hyun Jin Park, Jin Seo, Young Han Lee, Ho-Taek Song, Young Joo Min

**Affiliations:** 1 Biomedical Research Center, Ulsan University Hospital, University of Ulsan College of Medicine, Ulsan, Korea; 2 Department of Otorhinolaryngogly, Ulsan University Hospital, University of Ulsan College of Medicine, Ulsan, Korea; 3 Department of Pathology, Ulsan University Hospital, University of Ulsan College of Medicine, Ulsan, Korea; 4 Department of Biological Sciences, University of Ulsan, Ulsan, Korea; 5 Department of Radiology and Research Institute of Radiological Science, College of Medicine, Yonsei University, Seoul, Korea; 6 Division of Hematology-Oncology, Department of Internal Medicine, Ulsan University Hospital, University of Ulsan College of Medicine, Ulsan, Korea; Enzo Life Sciences, Inc., United States of America

## Abstract

KML001 is sodium metaarsenite, and has shown cytotoxic activity in human tumor cell lines. The anti-cancer mechanism of KML001 involves cancer cell destruction due to DNA damage at the telomeres of cancer cell chromosomes. In this study, we assessed the vascular disrupting properties of KML001 and investigated whether KML001 as VDA is able to increase anti-tumor activity in irinotecan combined treatment. We used a murine model of the CT26 colon carcinoma cell line. CT26 isograft mice treated intraperitoneally with 10 mg/kg KML001 displayed extensive central necrosis of tumor by 24 h. The vascular disrupting effects of KML001 were assessed by dynamic contrast enhanced magnetic resonance imaging. Gadopentetic acid-diethylene triaminepentaacetic acid contrast enhancement was markedly decreased in KML001-treated mice one day after treatment, whereas persistently high signal enhancement was observed in mice injected with saline. Rate constant K_ep_ value representing capillary permeability was significantly decreased (p<0.05) in mice treated with KML001. Cytoskeletal changes of human umbilical vein endothelial cells (HUVECs) treated with 10 uM KML001 were assessed by immune blotting and confocal imaging. KML001 degraded tubulin protein in HUVECs, which may be related to vascular disrupting properties of KML001. Finally, in the mouse CT26 isograft model, KML001 combined with irinotecan significantly delayed tumor growth as compared to control and irinotecan alone. These results suggest that KML001 is a novel vascular disrupting agent, which exhibits significant vascular shut-down activity and enhances anti-tumor activity in combination with chemotherapy. These data further suggest an avenue for effective combination therapy in treating solid tumors.

## Introduction

KML001 (sodium metaarsenite) is an orally available arsenic compound that has entered phase II clinical trials in solid tumors. This agent has shown cytotoxic activity in human tumor cell lines. Significant anti-cancer effects and stabilization have been reported in clinical studies with prostate cancer patients. KML001 was reported to be able to destroy and control cancer cells by causing DNA damage only at the telomeres of chromosomes in cancer cells [1]. However, such clinical results cannot be fully explained only with the aforementioned mechanisms because the agent shows anti-cancer effects in patients with a variety of cancers and is less toxic. Accordingly, information is needed concerning the main molecular biological mechanisms that may explain the anti-cancer effects of KML001.

Anti-angiogenic drugs applied in clinical trials as treatments for solid tumors including lung cancer and colorectal cancer exert their anti-cancer effects by normalizing the pathological abnormalities of tumor vessels and controlling neovascularization [2]. Among these drugs, vascular disrupting agents (VDAs) are a recent addition and represent a novel therapeutic approach. A VDA has different mechanism from existing anti-vascular endothelial growth factor (VEGF) agents such as bevacizumab, sunitinib and sorafenib. Combined with tubulin in the endothelial cells of the blood vessels, VDAs inhibit blood flow to tumors and cause tumor necrosis within a few hours after administration [3]. Because VDAs rarely affect normal vessels, they do not cause malfunction of liver, kidneys, brain or any other normal organs. Therefore, VDAs are regarded as safe and minimally toxic [4]. The selectivity of VDAs for tumor vessels is thought to be a consequence of the vessels’ pathological abnormalities. VDAs bind to the tubulin of the endothelial cells in the tumor vessels and cause cell-to-cell junctions or cytoskeleton disruption. They transform the endothelial cell shape, increase protein permeability in the blood vessels, and cause vascular occlusion by vascular constriction arising from increased interstitial pressure, increased blood viscosity, hemo-concentration and serotonin secretion [4]. This causes tumor hypoxia and necrosis. VDAs under development or under clinical trials include CA4DP, AVE8062A and OXi4503 [5]. In combination with the standard anti-cancer therapy in Phase II trials in patients with advanced lung cancer, ASA404 has extended survival [6].

Since arsenic was first shown to have clinical effects in patients with acute promyelocytic leukemia (APL) in China in the 1970s, more than 80% of patients treated with arsenic trioxide (ATO) have displayed beneficial clinical effects without acute toxicity [7], [8]. The biological activity of ATO reported so far explains the anti-cancer effects with a variety of mechanisms including anti-tubulin effect, differentiation induction, apoptosis, anti-proliferative activity and angiogenesis inhibition. [9]–[11]. Acute tumor vascular shutdown and massive tumor necrosis similar to those observed in VDAs was documented when ATO was administered in a murine tumor model [12]. Given that KML001 is a derivative of arsenic trioxide, it is very possible that the anti-cancer effect of the agent might result from tumor vascular disruption.

Here, we report the vascular disrupting effects of KML001 in CT26 isograft mice. The compound did not affect blood supply to normal organs including liver and kidneys, and so had limited effects on tumor vessels. Use of human umbilical vein endothelial cells (HUVECs) supported the view that KML001’s vascular disrupting effect resulted from the morphologic change of endothelial cells by cytoskeleton-associated protein degradation of tubulin. Our results indicate that KML001 is a new VDA, which, in combination with irinotecan, enhances anti-tumor activity in CT26 isograft mice.

## Materials and Methods

### Cell Culture and Reagents

HUVECs were purchased from ATCC (Manassas, VA, USA) and were maintained in Ham's Kaighn's Modification F12 (F12K; Invitrogen, Carlsbad, CA, USA) supplemented with 2 mM L-Glutamine (Invitrogen) and 0.1 mg/ml heparin sodium salt from porcine intestinal mucosa (Sigma-Aldrich, St. Louis, MO, USA), 0.05 mg/ml Endothelial Cell Growth Supplement (ECGS; BD, Franklin Lakes, NJ, USA) and 10% fetal bovine serum (FBS; Invitrogen). The medium was prepared fresh every 2 weeks. The murine CT26 colon carcinoma cell line (ATCC) was routinely maintained in RPMI 1640 medium supplemented with 10% FBS. KML001 (sodium metaarsenite, formerly known as Kominox) was obtained from Komipharm International (Seoul, Korea) and 50 mmol/l stock solutions were prepared in phosphate buffered saline (PBS). Aliquots of the stock were stored at −20°C. The stock solutions were stable for over 1 year. Working concentrations were freshly prepared daily by diluting the stock with complete RPMI 1640. Irinotecan (Sigma-Aldrich) was prepared at a 0.1 M concentration in PBS.

### Animal and Tumor Model

All research was governed by the principles of the Guide for the Care and Use of Laboratory Animals and approved by the University of Ulsan Animal Care and Use Committee. Female Balb/c mice (6–8 weeks of age) were obtained from ORIENTBIO (Seoul, Korea) and were maintained under specific pathogen-free conditions.

### Histological Analysis

CT26 cells were harvested from monoconfluent monolayer cell cultures in 1×PBS and 2×10^6^ cells in a total volume of 100 µl and were injected subcutaneously. When the tumor size became 3 mm in diameter, the control group was injected with PBS solution with 5% dextrose, while the experimental group was intraperitoneally injected with 100 µl of KML001 at a concentration of 10 mg/kg with 5% dextrose. At 8, 24 and 48 hours after injection, the liver, spleen and tumor tissues were acquired and put into a 37% solution of formaldehyde for 24 hours. The tissues were inserted into paraffin and sectioned at a thickness of 4 µm using a microtome (SLEE MAINZ GmbH, Germany). The sections were placed on slides and stained with Mayer's hematoxylin and eosin Y (both from Sigma-Aldrich). Images were observed using a model BX50 inverted microscope (Olympus, Tokyo, Japan).

### Tumor Perfusion Measurement

Magnetic resonance imaging (MRI) examination was performed 8 and 9 days after subcutaneous inoculation of 2×10^6^ CT26 cells. Balb/C mice were anesthetized by 2% isoflurane mixed with 100% 1 l/min O_2_ via a nose cone. A catheter was inserted into the tail vein for injection of 281 mg/kg gadopentetic acid-diethylene triaminepentaacetic acid (Gd-DTPA; Magnevist, Schering, Germany). T2-weighted turbo spin echo images were acquired on coronal planes using the following parameters: TE = 60 ms, TR = 3200 ms, field of view (FOV) = 50 mm, RFOV (%) = 69.94%, matrix scan = 224, reconstruction = 512, 0.5 mm thick slices, 14 slices, and acquisition time = 8 min 38 s. T1-weighted spin-echo images were acquired on coronal planes as pre- and post-dynamic contrast enhanced MRI scan using the following parameters: TE = 7.9 ms, TR = 427 ms, field of view (FOV) = 50 mm, RFOV (%) = 70%, matrix scan = 128, reconstruction = 256, 1 mm thick slices, 11 slices, and acquisition time = 2 min 8 s. For dynamic contrast enhanced (DCE)-MRI scan, Gd-DTPA (Magnevist) was administrated at a dose of 281 mg/kg via tail-vein injection. DCE-MRI was performed using a perfusion-weighted spin echo sequence using the following acquisition parameters: FOV (mm) = 50×35, RFOV (%) = 71, matrix scan = 112, reconstruction = 224, TR/TE (ms) = 12/4.0, slice number = 11, dynamic scans = 60, 1 mm slice thickness with a total acquisition time of 2 min 6 s.

MRI images were downloaded from a Philips MRI scanner in the Philips PARREC format, and the images were transferred to a processing workstation connected to the research network. The programming languages used were IDL 8.1 (ITT, Boulder, CO, USA). The data was analyzed by the BRIX model method.

### Cytotoxicity Assay

The cytotoxicity assay was used to determine drug-mediated cytotoxicity by using (3-(4,5-dimethylthiazol-2-yl)-5-(3-carboxymethoxyphenyl)-2-(4-sulfophenyl)-2H-tetrazolium) (MTS) reagent of the Cell Titer 96 Aqueous One Solution (Promega, Madison, WI, USA) with absorption measured at 490 nm, as described previously [16]. Briefly, the assay entailed seeding HUVECs at 0.5×10^5^ cells per well for 24 h at 37°C and 5% CO_2_. After addition of the drugs at the indicated concentrations, the cells were incubated for either 24 or 48 h. Twenty microliters of the MTS solution was added to each well and incubated at 37°C for 4 h. Thereafter, absorbance values (A) for each well were measured using a microplate spectrophotometer (Bio-Tek, Winooski, VT, USA). The percentage of viable cells was calculated using the background-corrected absorbance using the following calculation: % cytotoxicity = (1–A of experimental well/A of positive control well) × 100.

### Flow Cytometry

HUVECs (2×10^6^ cells) were treated with each drug at the indicated concentration for 24 h. Cells were washed with PBS by spinning at 1,000 rpm for five min at 4°C and fixed with 1 ml of ice-cold 95% ethanol drop-wise while vortexing. The fixed cells were incubated on ice for at least 30 min and then washed as described above. The pellets were resuspended in 1 ml PBS and 100 µl of a 1 mg/ml propidium iodide (PI) stock was added for 5–10 min at room temperature. Flow cytometry was performed using a FACSCalibur flow cytometer and Cell Quest software (BD Biosciences).

### Immunoblots

HUVECs grown to 70%–80% confluence in a F25 culture flask were harvested after drug treatment in 100 µl/well of protein lysis buffer containing 50 mM Tris, pH 7.5, 150 mM NaCl, 1% NP-40, 0.5% sodium deoxychloate, 0.1% sodium dodecyl sulfate (SDS), and protease inhibitor (protease inhibitor cocktail tablets; Roche, Basel Switzerland) plus 200 units/ml aprotinin (Sigma-Aldrich) and 10 µmol/l trichostatin A (Cayman Chemical, Ann Arbor, MI, USA). Protein concentrations were determined using a commercial protein assay reagent (Bio-Rad, Hercules, CA, USA) and 3 µg protein were loaded into each well and resolved using 10% SDS-polyacrylamide gel electrophoresis (SDS-PAGE). After protein transfer onto nitrocellulose, blots were blocked using 5% milk and each blot was probed with anti-glyceraldehyde 3-phosphate dehydrogenase (GAPDH) (Santa Cruz Biotechnology) as an internal control. Other primary antibodies used were anti-α-tubulin (Santa Cruz Biotechnology) and the membrane was reacted with goat anti-mouse lgG secondary antibody (Santa Cruz Biotechnology) conjugated to horseradish peroxidase (HRP) and exposed to light with Molecular Imager ChemiDoc XRS (Bio-Rad) using Immun-Star™ and quantified by Quantity One Image analyzer (Bio-Rad).

### Reverse Transcription-polymerase Chain Reaction (RT-PCR)

HUVECs (ATCC) were cultivated in F25 culture flasks containing F-12K medium (Sigma-Aldrich) supplemented with 10% FBS and ECGS (Sigma-Aldrich). After cultivation for 24 h, the cells were treated with 5 uM, 10 uM and 20 uM KML001. At 6 and 48 h after the treatment, the cells were collected. Total RNA was extracted using Trizol (Invitrogen) from the collected cells using alcohol precipitation. One microgram of total RNA was reverse transcribed. PCR was performed on cDNA template using Taq polymerase (Bioneer, Seoul, Korea) and the following primers: α-tubulin (sense: 5′-ATT GTG CCT TCA TGG TAG AC-3′, antisense: 5′-TTC TGT CAG GTC AAC ATT CA-3′), β-tubulin (sense: 5′-AAC GAC CTC GTC TCT GAG TA-3′, antisense: 5′-AAT TCT GAG GGA GAG GAA AG-3′) and GAPDH (sense: 5′-ACC ACT TTG TCA AGC TCA TT-3′, antisense: 5′-AGT GAG GGT CTC TCT CTT CC-3′). The PCR product was confirmed by 1.5% agarose gel electrophoresis.

### Immunofluorescene and Confocal Microscopy

HUVECs were cultured on the confocal dish (SPL, Seoul, Korea) with F-12K (Sigma-Aldrich) medium containing 10% FBS and endothelial cell growth supplement (ECGS, E2759; Sigma-Aldrich). After cultivation for 24 h, the cells were treated with KML001 at concentrations of 1 uM, 5 uM, or 10 uM. At 24 h and 48 h after the treatment, the cells were fixed with 3.7% paraformaldehyde at the room temperature for 10 min. After being washed with PBS solution three times, the cells were treated with 0.15% Triton X-100 for 15 min and washed three times with PBS. After being blocked with 2% bovine serum albumin (BSA, Sigma-Aldrich) at room temperature for 60 min, the cells were also washed three times with PBS. Monoclonal antibody against α-tubulin (Santa Cruz Biotechnology, Santa Cruz, CA, USA) was used to react with cell tubulin at room temperature for 1 h and the cells were washed three times with PBS. They were reacted with rat anti-mouse IgG1 secondary antibody (BD Bioscience, Franklin Lakes, NJ, USA) conjugated to fluorescein isothiocynate (FITC). After being washed three times with PBS, the cells were analyzed using a model FV500 confocal laser scanning microscope (Olympus, Tokyo, Japan).

### Anti-tumor Activity

Anti-tumor effects of irinotecan or KML001 were evaluated in the mouse CT26 isograft model. Female BALB/c mice weighing approximately 20 g were inoculated by subcutaneous injection with 2×10^6^ of CT26 cells per mouse. When tumors became 3 mm in diameter, the control group was injected weekly with PBS containing 5% dextrose. The experimental groups were divided into a group injected with 100 µl of irinotecan (15 mg/kg/week with 5% dextrose, for 4 weeks) and the other group injected with 100 µl of both KML001 (10 mg/kg/week with 5% dextrose, for 4 weeks). Mice were monitored for toxicity by body weight measurements and tumor growth was measured once every 2 days using calipers throughout the experimental period. Tumor volumes were calculated based on the following formula: tumor volume = (length×width^2^)×π/6.

In a combination experiment, when the tumor size reached 3 mm in diameter, mice were randomized into different treatment groups: irinotecan alone (15 mg/kg/week with 5% dextrose), irinotecan (15 mg/kg) combined with KML001 (10 mg/kg), and vehicle control (1×PBS with 5% dextrose). All experiment groups were treated weekly for 4 weeks.

### Statistical Analysis

The results obtained from at least three independent experiments were expressed as mean ± standard deviation. One-way ANOVA test were used to determine the differences between control and treatment groups. P<0.001 was considered to be statistically significant. All data were analyzed using GraphPad software.

## Results

### Gross Morphological and Histopathological Changes

**Figure 1:**
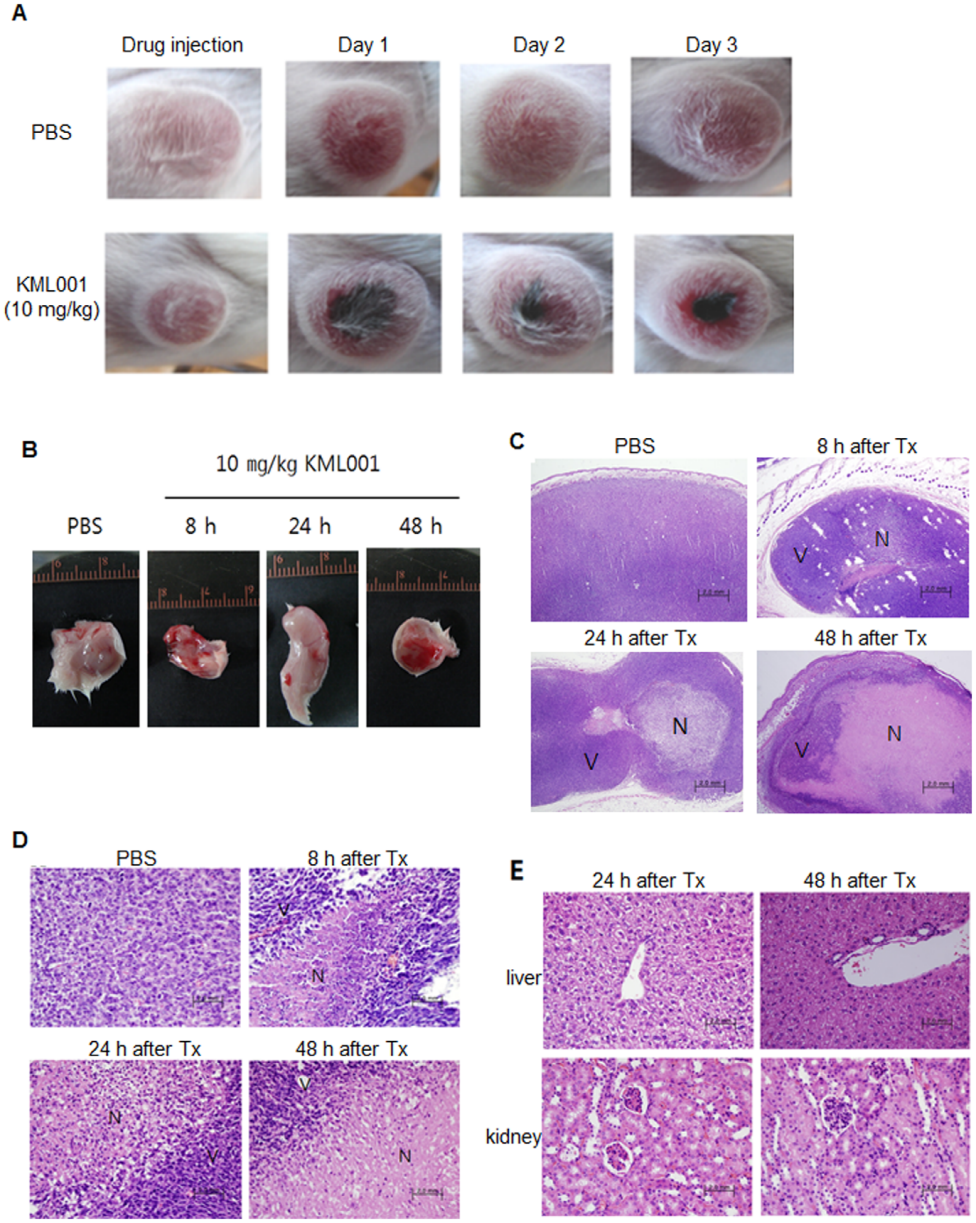
Gross morphological and histopathological changes. (A) Observation on necrosis in tumor after treatment of KML001 in CT26 isograft model. (B) Tumors were excised from CT26 isografts treated with PBS or KML001. (C–E) Histological analysis of tumor tissues (C, x100 and D, x400) and normal tissues (E) treated with KML001.

Fig. 1A illustrates the gross tumor morphology before and after a single injection ofKML001. While the discoloration of the tissues of the mice injected without KML001 was not observed for a certain period of time, the central part of tumors in the tissues of the mice injected with KML001 became discolored at 24 h after the injection of KML001. With time, the tumors became discolored regardless of the injection of KML001. In case of white mice injected with KML001, the tissues in the central part became discolored by necrosis.

The sequential histopathological changes after drug treatment are shown in Fig. 1B and 1C. In case of the tumor tissues of white mice who did not receive KML001, the cell density remained constant and the cells were distributed without any change in shape. On the contrary, when the exposure time of KML001 increased, the scope of necrotic cell death in the tumor tissues of mice injected with KML001 increased and necrotic cell death was evident in the central part of the tumors. In liver, spleen and kidney tissues, KML001-mediated ischemic damage was not observed.

### Quantitative Analysis of DCE-MRI

**Figure 2:**
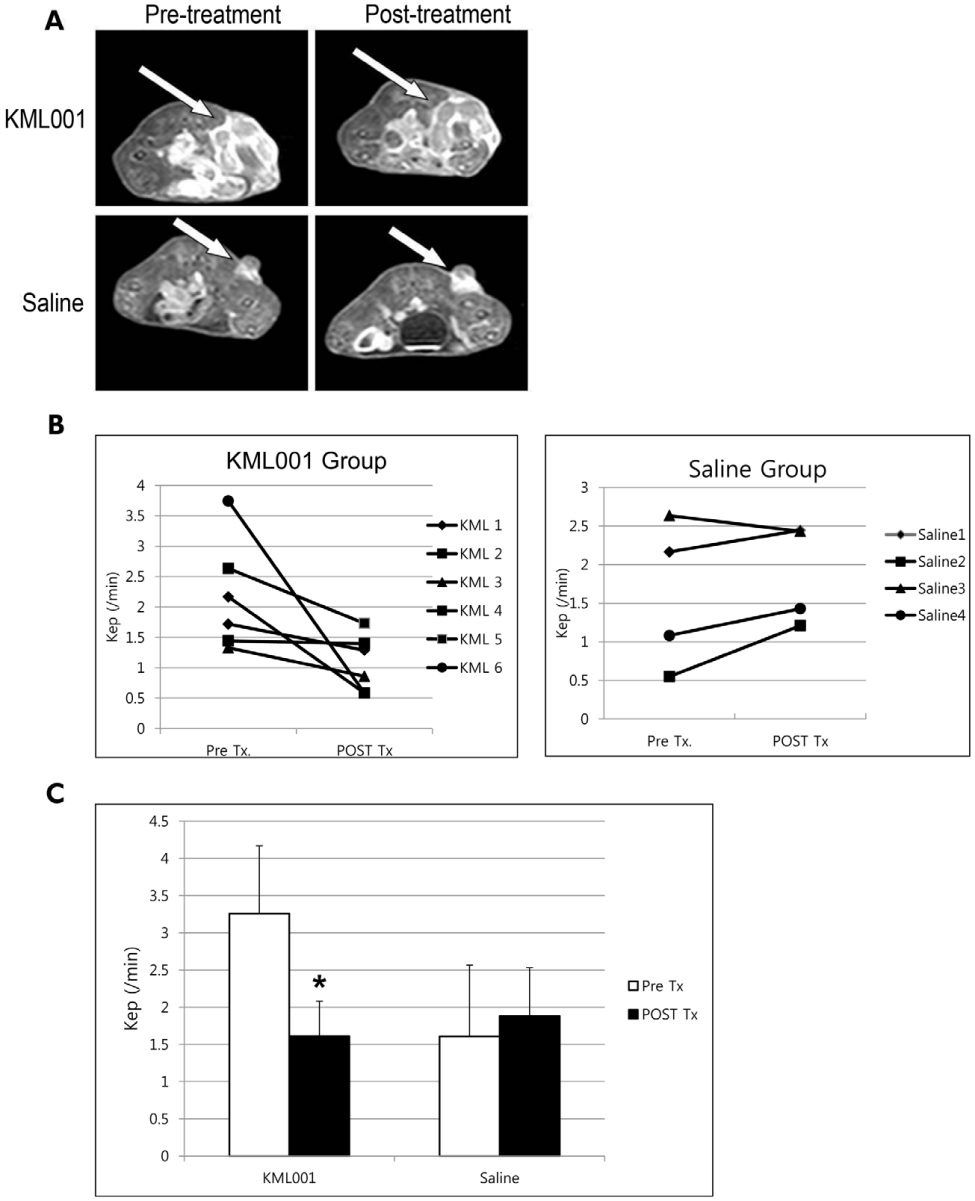
Quantitative analysis of the DCE-MRI parameter in the CT26 isograft model. (A) T1-weighted gadolinium contrast enhanced MRI of pre-treatment and 24 h post-treatment. Arrows indicates enhancing tumors at the proximal hind leg of the mice. (B) The quantitative parameter K_ep_ values of each animal treated with KML001 and saline. (C) Changes in the mean values of K_ep_ measured pre and 24 h post-treatment. (*indicate P<0.05).

T1-weighted MRI revealed a difference between pre-treatment and 24 h post-treatment Gd-DTPA contrast enhancement (Fig. 2A). The degree of enhancement was significantly decreased in the KML001 treated group as compared to the saline injection group. Fig. 2B shows the decreasing of the quantitative parameter K_ep_ values of each animal treated with KML001 and saline. K_ep_ representing vascular leakage was significantly decreased in the KML001 treatment group at 24 h after injection (Fig. 2C).

### Analysis of Apoptosis and Cytotoxicity of HUVECs by KML001

**Figure 3:**
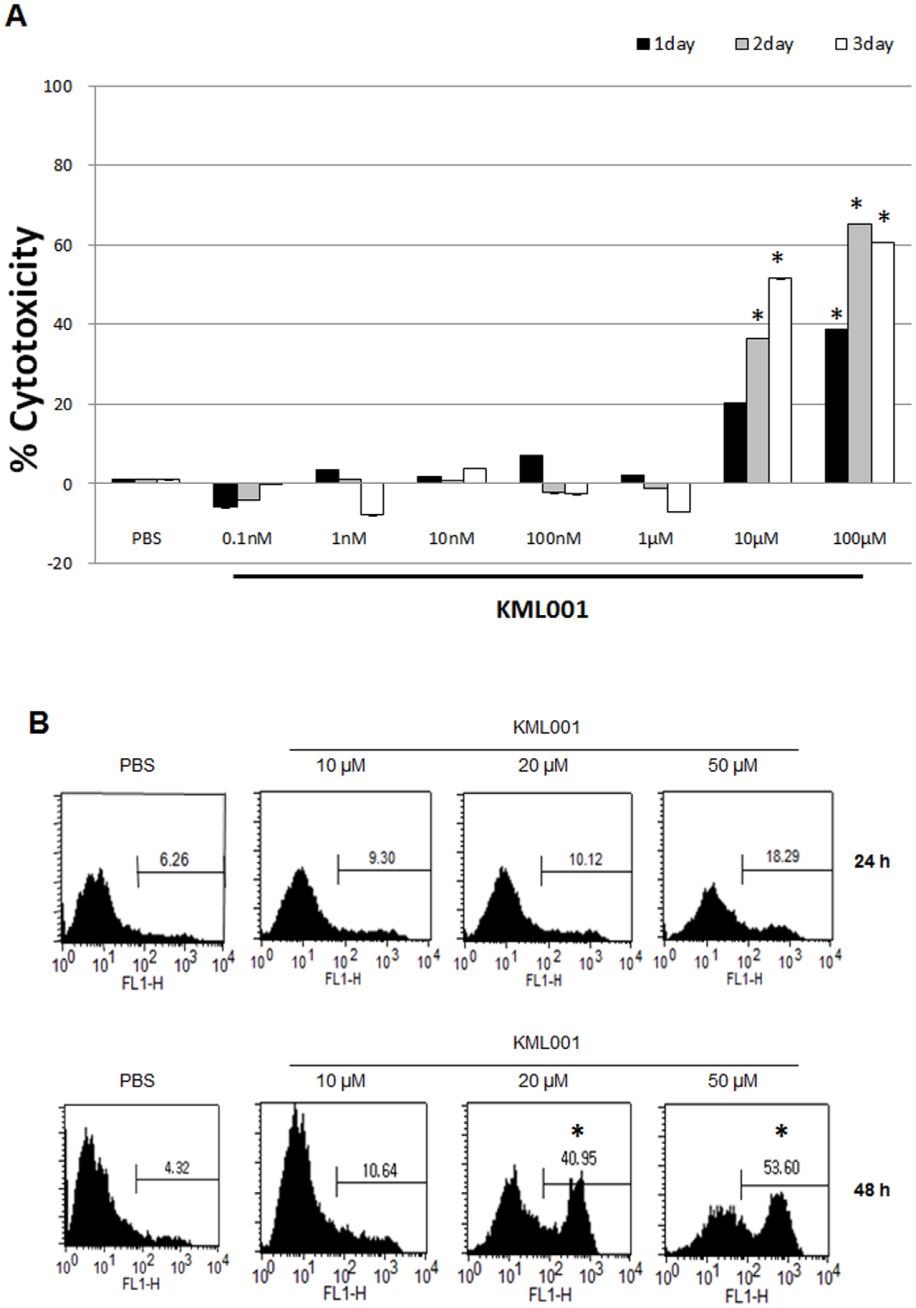
Analysis of apoptosis and cytotoxicity of HUVECs by KML001. (A) Effects of KML001 on the cytotoxicity of HUVECs Cell cytotoxicity was assessed by the MTT assay. Representative of three independent experiments. Columns, mean; bars, SD. (*indicate P<0.05 compare to PBS) (B) Induction of apoptosis in HUVECs by KML001. (*indicate P<0.05 compare to PBS).

To assess the effect of KML001 on vascularization, we first analyzed the effect on endothelial cells, which are important for vascularization. Cytotoxicity was evident at 10 uM and was induced more in proportion to time (Fig. 3A). In addition, KML001 as an arsenic derivative is known to cause a variety of physiologic effects. Fluorescence-activated cell sorting analysis revealed that apoptosis increased depending on the concentration of KML001, and was especially apparent with prolonged treatment (Fig. 3B).

### KML001 Induces Microtubule Depolymerization in HUVECs

**Figure 4:**
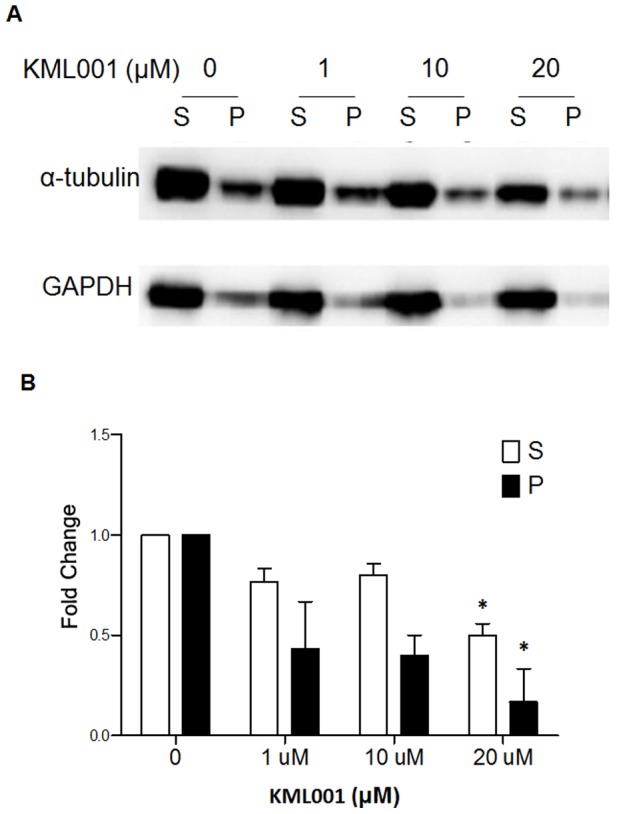
KML001 induces depolymerization of microtubules in HUVECs. HUVECs were treated with KML001, or no drug as the control, for 24 h at the indicated concentrations. (A) Cell lysates were separated into polymerized (P) or soluble (S) fractions by centrifugation 2000 rpm at 22°C for 30 min. Equal volumes were separated by SDS-PAGE and evaluated by Immunoblot probed with anti-α-tubulin. (B) Densitometric analysis showing relative protein level of each fraction and normalized with respect to GAPDH are shown. Representative of three independent experiments. Columns, mean; bars, SD. (*indicate P<0.05 compare to 0 µM).

To investigate whether apoptosis was caused by a cytoskeletal abnormality, which is important for cell division and cell differentiation, the change in α-tubulin expression in the cytoskeleton was identified by the division of tubulin into the isolated form (soluble, S) and polymerized form (ppt, P). This procedure and subsequent modifications have been widely used and allow a quick and consistent assessment of the proportion of tubulin polymer in cells under a variety of experimental conditions. An increase in the S fraction served as an indicator of destabilized tubulin. A higher concentration of KML001 reduced the protein level in both the supernatant liquid (isolated tubulin) and the sediment (polymerized tubulin) (Fig. 4A and 4B). The findings were consistent with the notion that KML001 not only isolates the polymerized tubulin from the polymer but also reduces the overall amount of tubulin by inducing the isolated tubulin.

### Change in Tubulin Protein in HUVECs Treated with KML001

**Figure 5:**
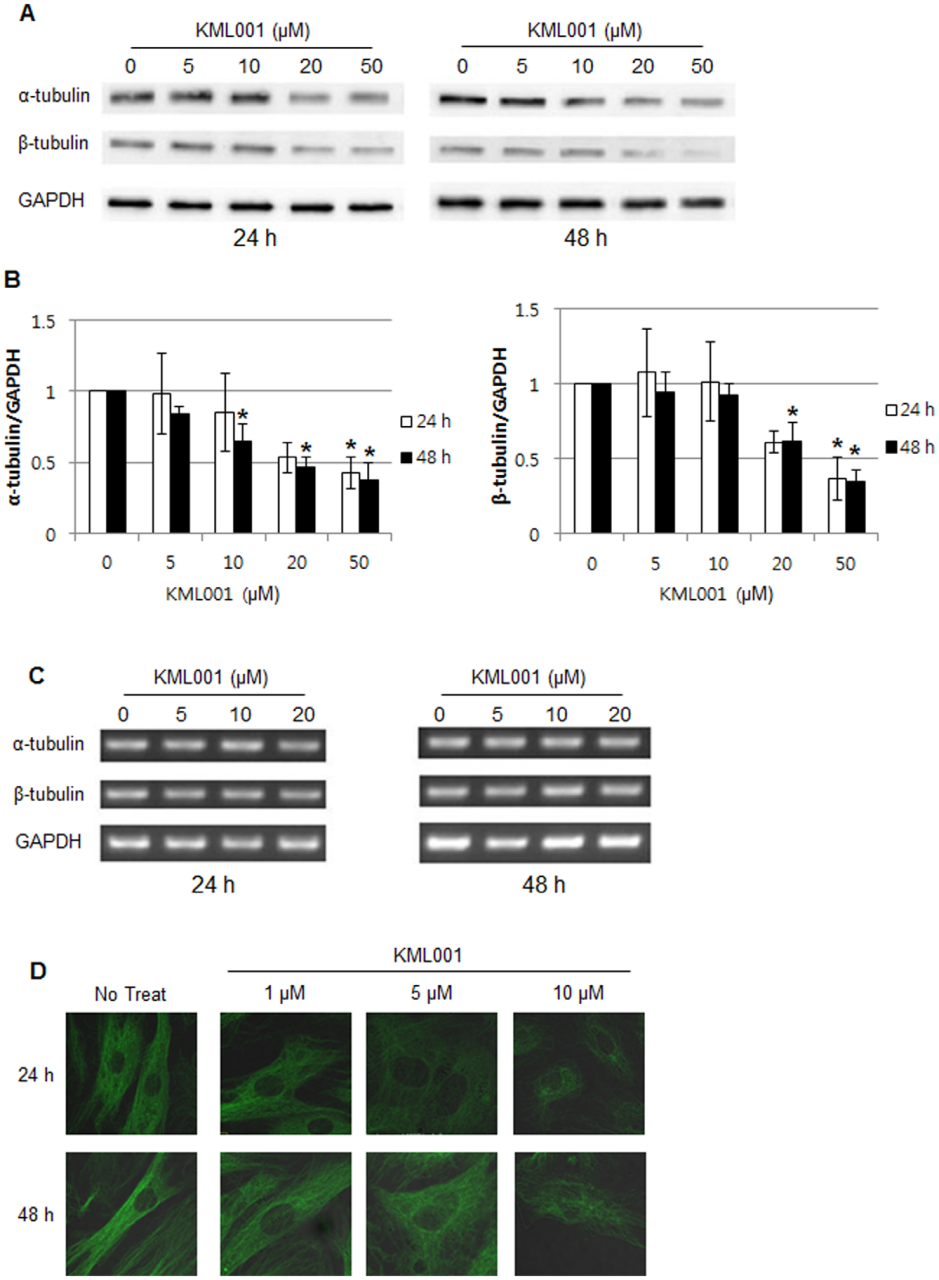
KML001 promotes degradation of α- and β-tubulin in HUVECs. (A) Immunoblot of HUVECs treated with KML001 (0, 5, 10, 20, and 50 µM) for 24 h and 48 h. Blots were hybridized with α- and β-tubulin antibody. GAPDH was used as a loading control, (B) Densitometric analysis showing relative protein level of α-tubulin and β-tubulin and normalized with respect to GAPDH are shown. Representative of three independent experiments. Columns, mean; bars, SD. (*indicate P<0.05 compare to 0 µM). (C) Tubulin mRNA expression in HUVECs treated with KML001 (0, 5, 10, and 20 µM) for 24 h and 48 h. (D) Analysis of change in microtubules by immunocytochemistry from HUVECs treated with KML001.

To check the change in the overall expression of tubulin, the total amount of tubulin protein was identified by Western blotting. A higher concentration of KML001 reduced the protein level on α-tubulin and β-tubulin (Fig. 5A and 5B), supporting the idea that KML001 drives the specific destruction of α-tubulin and β-tubulin proteins.

To make it clear whether the change in tubulin protein resulted from the cellular gene expression level through the aforementioned experiment or not, RT-PCR was performed. α-Tubulin and β-tubulin mRNA levels were unchanged at 24 h and 48 h after the treatment of KML001 at each concentration (Fig. 5C). Because KML001 treatment resulted in the destabilization of tubulin, we tested whether KML001 treatment affected the cellular microtubule network. Cells treated with KML001 displayed a reduced total amount of microtubules, depending on the KML001 concentration (Fig. 5D). These data supported the suggestion that the quantitative change in the protein of α-tubulin and β-tubulin from HUVECs was not caused by the change in the expression of mRNA, but by the change in the protein level.

### Anti-tumor Activity of KML001 in the CT26 Isograft Model

**Figure 6:**
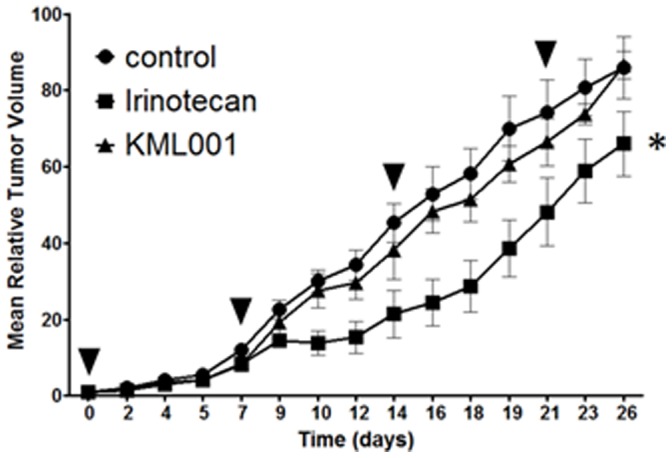
KML001 alone treatment has a no inhibitory effect on tumor in CT26 isograft models. Control group mice were injected intraperitoneally with saline. Irinotecan group mice were injected with 15 mg/kg irinotecan. KML001 group was injected with 10 mg/kg KML001. All experiment groups were administrated once weekly for 4 weeks. Black arrowheads indicate treatment. *P<0.0001 compared to control.

Because irinotecan (CPT-11) is a chemotherapeutic agent widely used in colorectal cancer, small cell lung cancer and several other solid tumors [13], we choose irinotecan as a combination partner in this study. To evaluate the anti-tumor effects of irinotecan alone or KML001 alone, CT26 isograft mice were treated with KML001 (10 mg/kg) or irinotecan (15 mg/kg), and tumor growth was compared with vehicle-treated control. KML001 alone treatment did not delay the progression of tumor growth, but irinotecan alone delayed progression of tumor growth to day 26 (Fig. 6). This data suggested that KML001 alone treatment has a no inhibitory effect on tumor in the CT26 isograft model.

### Irinotecan Combined with KML001 has an Additive Inhibitory Effect in the CT26 Isograft Model

**Figure 7:**
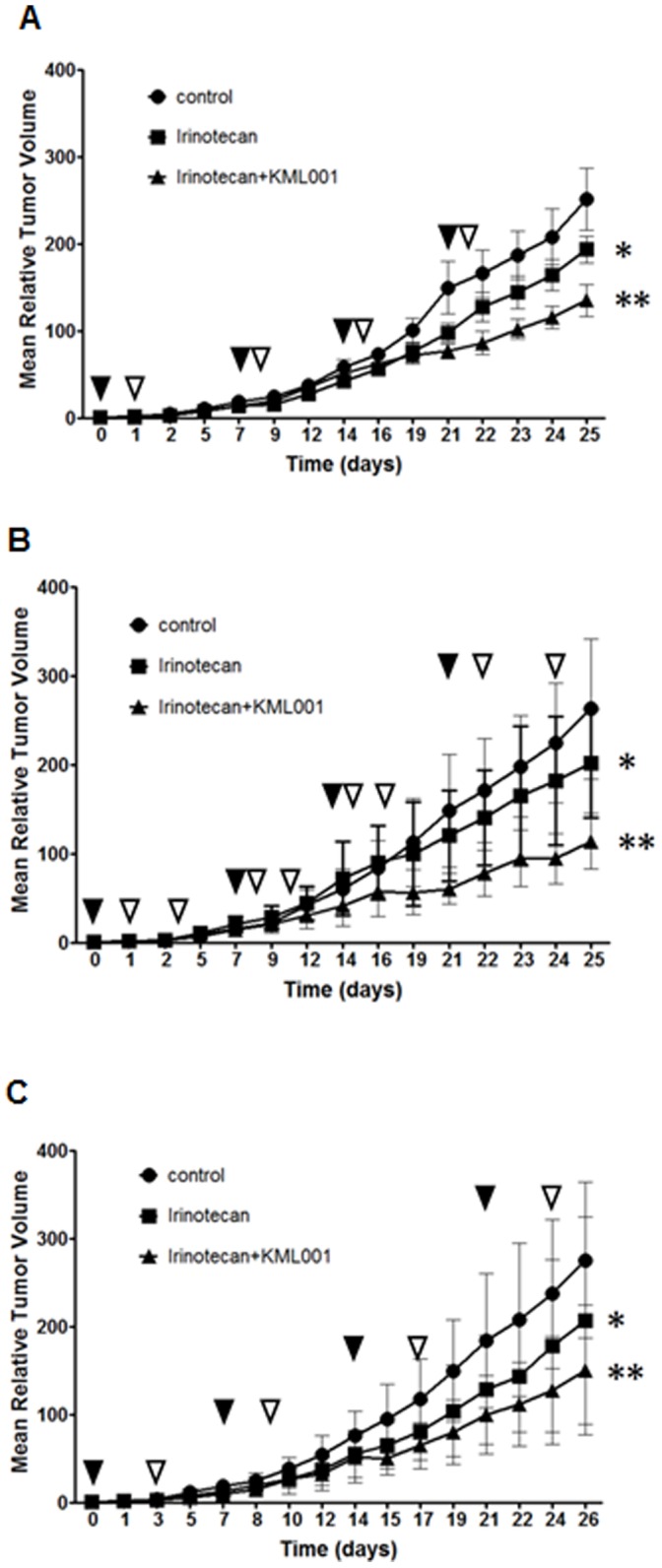
Irinotecan combined with KML001 has an additive inhibitory effect in CT26 isograft models. (A) At 24 h after being injected with irinotecan (once weekly), injected with KML001 (once weekly). (B) At 24 h and 72 h after being injected with irinotecan (once weekly), injected with KML001 (twice weekly). (C) At 72 h after being injected with irinotecan (once weekly), injected with KML001 (once weekly) and all experiment groups were performed for 4 weeks. Black (irinotecan) and white (KML001) arrowheads indicate treatment. *, P<0.0001 compared to control. **, P<0.0001 compared to irinotecan.

To evaluate the anti-tumor effects of irinotecan treatment alone or in combination with KML001, we used four different sequences of administration. The experimental groups were divided into one group injected with 100 µl of irinotecan at the concentration of 15 mg/kg and a second group injected with 100 µl of KML001 (10 mg/kg in 5% dextrose) and irinotecan (15 mg/kg) at defined times. Twenty four hours after being injected with irinotecan, mice were injected with KML001 (Fig. 7A). At 24 h and 72 h after being injected with irinotecan, mice were injected with KML001 (Fig. 7B). At 72 hours after being injected with irinotecan, mice were injected with KML001 (Fig. 7C). All treatments were administrated once weekly for 4 weeks. Compared to the administration of only irinotecan, KML001+ irinotecan produced a 2-fold increase in tumor inhibition.

In the CT26 isograft model, growth was inhibited with irinotecan alone as compared to control (23.0%, 23.2%, and 25.0% in Fig. 7A, 7B, and 7C, respectively). However, mice treated with KML001 + irinotecan showed a significantly tumor growth delay as compared to control and irinotecan alone (45–56%).

## Discussion

VDAs have shown much promise pre-clinically as anti-cancer therapeutics, and a small number are currently being investigated in clinical trials. However, the failure of such agents to target the peripheral tumor rim means that their efficacy as a single-agent therapeutic strategy is in need of improvement [14]. One revised strategy involves treatment in combination with standard chemotherapeutic agent that can destroy the remaining tumor cells. There have been several pre-clinical studies that have demonstrated improved efficacy [15]. This approach has recently been reported as having some success in Phase II clinical trials using combretastatin A4 phosphate in combination with carboplatin and paclitaxel [16]. In this study, we demonstrate that KML001 induces a prompt and selective vascular shut down leading to massive central necrosis in CT26 isograft model.

The biological response of tumors to VDA treatment is typically characterized by early increases in vascular permeability followed by vascular collapse and cessation of blood flow leading to ischemia and tumor necrosis [17]–[19]. DCE-MRI is one of the most widely used imaging methods for assessment of angiogenesis in preclinical studies [20], [21]. Several studies have highlighted the usefulness of MRI in the assessment of tumor vascular response to VDAs [22]. In this study, we also demonstrated the vascular effect of KML001 using DCE-MRI. T1-weighted MRI revealed a difference between pre-treatment and 24-h post-treatment Gd-DTPA contrast enhancement. Degree of enhancement was significantly decreased in the KML001 treated group compared to the saline injection group. K_ep_ representing vascular leakage was significantly decreased in KML001 treatment group at 24 h after injection.

First introduced into clinical oncology in the 1960s, microtubule target agents (MTAs) are essential components in the therapy of many cancers, including lymphoma as well as breast, ovarian, lung, and head and neck cancers [23]. In cancer cells, the focus has often been on their ability to interfere with mitosis, a thesis developed with rapidly proliferating in vitro models that has never been proven in patients [24]. Tubulin polymerization inhibitors act primarily by disrupting the tubulin network of the endothelial cell cytoskeleton, leading to shape changes and increased vascular permeability. Our in vitro study results provide supportive evidence of increased tumor vascular damage following KML001 treatment. KML001 reduced the protein level on both the supernatant liquid (isolated tubulin) and the sediment (polymerized tubulin) by immunoblot using HUVECs and then reduced the total amount of microtubules depending on concentration, as shown by confocal imaging. These results support that KML001 is a novel VDA.

Furthermore, we evaluated the anti-tumor efficacy of irinotecan in combination with KML001. The sequence of administration should be carefully designed to avoid an effect of one agent with the other. Ideally, a combination of VDAs and cytotoxic agents is expected to take advantage of the effect of the former on endothelial cells and of the latter on tumor cells. The effects of VDAs on the vasculature have obvious important implications in the design of combination treatments with these agents, given their possible interference with the distribution of the cytotoxic drug [25]–[27]. In this respect, the sequence of administration can be selected according to two main rationales: on the one hand, vessel shutdown induced by the VDA given after the cytotoxic compound would cause trapping of the already present cytotoxic drug within the tumor, and, at the same time, would prevent the possible VDA-induced impairment of drug distribution in the tumor. So, in this study, we used the three different sequences of administration. Interestingly, all of different sequences showed the inhibitory effect of tumor growth more than the irinotecan alone group. In the CT26 isograft model, tumor growth was inhibited with irinotecan alone as compared to control (23.0%–25.0%). However animals treated with irinotecan+KML001 showed significant tumor growth delay as compared to control and irinotecan alone (45%–56%).

This study demonstrates that KML001 is a novel VDA, which exhibits significant vascular shut down activity in CT26 isograft model and enhances antitumor activity in combination with chemotherapy, and suggests a avenue for effective combination therapy in treating solid tumors.
